# Paragonimiasis Following Ingestion of Frozen Freshwater Crab: Two Case Reports

**DOI:** 10.1002/rcr2.70631

**Published:** 2026-05-27

**Authors:** Asuka Yamamura, Muneyuki Sekiya, Yusuke Usui, Susumu Sakamoto, Keiya Watanabe, Kohshi Fukuda, Yasuko Kurose, Naobumi Tochigi, Mio Kokubo, Haruhiko Maruyama, Kazuma Kishi

**Affiliations:** ^1^ Department of Respiratory Medicine Toho University Omori Medical Center Tokyo Japan; ^2^ Department of Surgical Pathology Toho University Omori Medical Center Tokyo Japan; ^3^ Department of Parasitology, Division of Infectious Diseases, Faculty of Medicine Miyazaki University Miyazaki Japan

**Keywords:** eosinophilic pneumonia, *Paragonimus westermani*, pleural effusion, pneumothorax, praziquantel

## Abstract

Paragonimiasis is a zoonotic disease caused by trematodes belonging to the genus *Paragonimus*. It is acquired by ingesting freshwater crustaceans or raw game meat. Here, we report two patients (a couple) who developed paragonimiasis and pulmonary lesions with peripheral eosinophilia after sharing and consuming previously frozen freshwater crabs (*Eriocheir japonica*) together at the same restaurant in Tokyo. One patient developed peripheral airspace consolidation, which was initially misdiagnosed as chronic eosinophilic pneumonia, whereas the other exhibited eosinophilic pleural effusion. Antiparasitic antibody testing of serum using enzyme‐linked immunosorbent assay (ELISA) established a diagnosis of paragonimiasis. Both cases were effectively treated with praziquantel. These cases demonstrate that frozen freshwater crabs remain a potential source of paragonimiasis, underscoring the critical role of dietary history in patients with eosinophilic lung diseases.

## Graphical abstract

Paragonimiasis is a zoonotic trematode infection acquired through ingestion of freshwater crustaceans or raw game meat. Here, we report two patients (a couple) who developed paragonimiasis after consuming previously frozen freshwater crabs together at the same restaurant in Tokyo. Both cases presented with pulmonary lesions and eosinophilia; one was initially misdiagnosed as chronic eosinophilic pneumonia, whereas the other developed eosinophilic pleural effusion. Serological testing confirmed the diagnosis. Our reported cases highlight the persistent risk associated with frozen freshwater crabs and the importance of a detailed dietary history in patients with pulmonary lesions and eosinophilia.

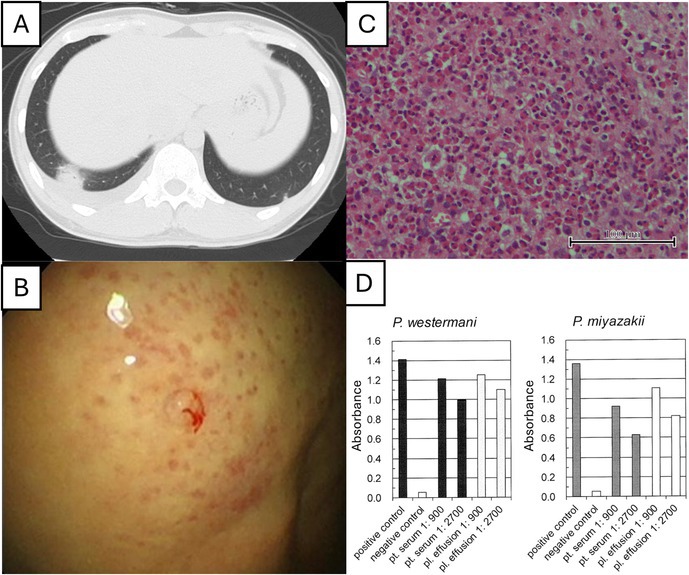

## 1 Introduction

Paragonimiasis is a zoonotic parasitic disease caused by trematodes of the genus *Paragonimus*, most commonly *Paragonimus westermani*. Humans typically acquire the infection by accidentally ingesting metacercariae, either through consumption of undercooked freshwater crustaceans or raw meat from wild animals such as wild boar and deer. Clinical manifestations vary with the site of parasite invasion. Imaging studies are crucial in identifying pulmonary abnormalities, such as airspace consolidation, nodular lesions, pneumothorax or pleural effusion. Peripheral eosinophilia is a hallmark laboratory finding [1]. Preventive measures that have been proven effective include thoroughly cooking or appropriately freezing freshwater crabs harbouring metacercariae [2]. Nevertheless, although freezing freshwater crabs is generally known to inactivate metacercariae and thereby prevent infection, this report describes two patients who developed paragonimiasis after consuming previously frozen freshwater crabs together at the same restaurant in Tokyo, each presenting with distinct clinical courses.

## 2 Case Report

### 2.1 Case 1

A 24‐year‐old Japanese woman had been experiencing palpitations for 2 weeks. Chest high‐resolution computed tomography (HRCT) revealed peripheral airspace consolidation surrounded by ground‐glass opacity in the right lower lobe. Blood tests showed marked peripheral eosinophilia (6572/μL) and elevated C‐reactive protein (CRP) levels (4.64 mg/dL). Chronic eosinophilic pneumonia (CEP) was suspected, for which oral corticosteroid therapy (prednisolone 25 mg/day) was initiated, resulting in a slight improvement. However, new pulmonary nodules developed during steroid tapering. Consequently, the patient was referred to our hospital after 6 weeks of corticosteroid therapy.

**FIGURE 1:**
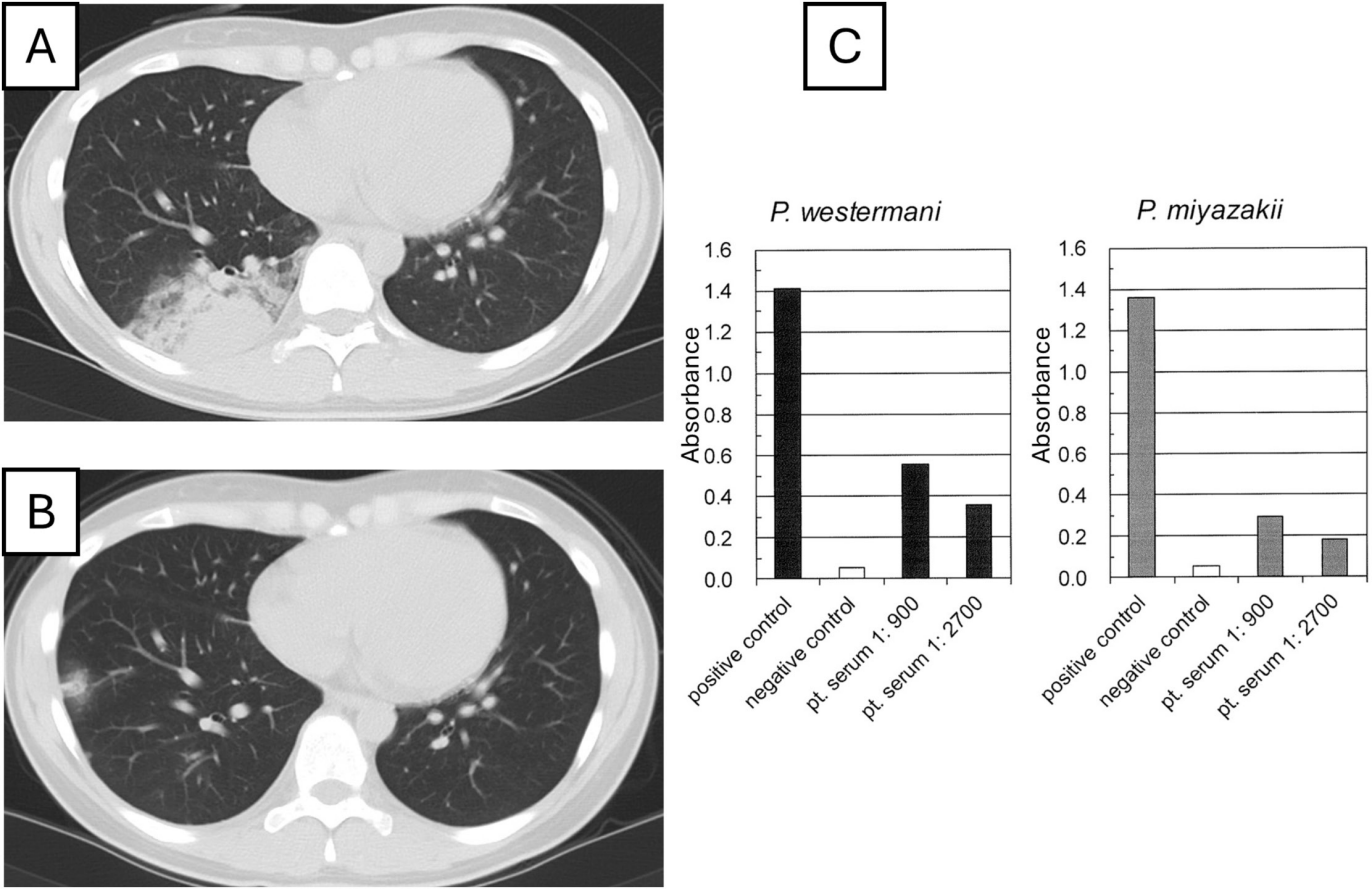
High‐resolution computed tomography (HRCT) images of Case 1 and results of antiparasitic antibody testing (microplate enzyme‐linked immunosorbent assay). (A) HRCT image obtained at the initial visit to the referring physician showing peripheral airspace consolidation in the right lower lobe. (B) At the time of initial presentation to our hospital, the consolidation showed partial resolution, whereas new nodules were observed in the right lower lobe. (C) Pretreatment serum antiparasitic antibody testing was positive for *Paragonimus westermani* and *P. skrjabini miyazakii*.

At the time of her initial visit to our hospital, the patient presented with peripheral eosinophilia (1496/μL) and mildly elevated CRP levels (2 mg/dL). Serum total IgE levels were normal, and biochemical and autoimmune tests were unremarkable. While airspace consolidation improved on HRCT, new nodules had developed in the right lower lobe (Figure 1A,B). Detailed history‐taking revealed that the patient had consumed previously frozen freshwater crabs (*Eriocheir japonica*) at a restaurant with Case 2 in Tokyo, 2 weeks before the onset of symptoms. Stool and sputum examinations were negative for parasite eggs.

Serum antiparasitic antibodies were assessed using a multiple‐dot enzyme‐linked immunosorbent assay (ELISA), which yielded positive results for *P. westermani* and *P. skrjabini miyazakii*. Seven weeks after her initial hospital visit, paragonimiasis was diagnosed, based on the patient's dietary history and microplate ELISA binding patterns (Figure 1C). Simultaneously, the patient developed mild left pneumothorax, which was managed conservatively. Five days after pneumothorax developed, oral praziquantel (75 mg/kg/day) was administered for 3 days, resulting in the improvement of pneumothorax, pulmonary lesions and laboratory findings. Follow‐up serology 4 months later showed decreased antibody levels, with no recurrence.

### 2.2 Case 2

**FIGURE 2:**
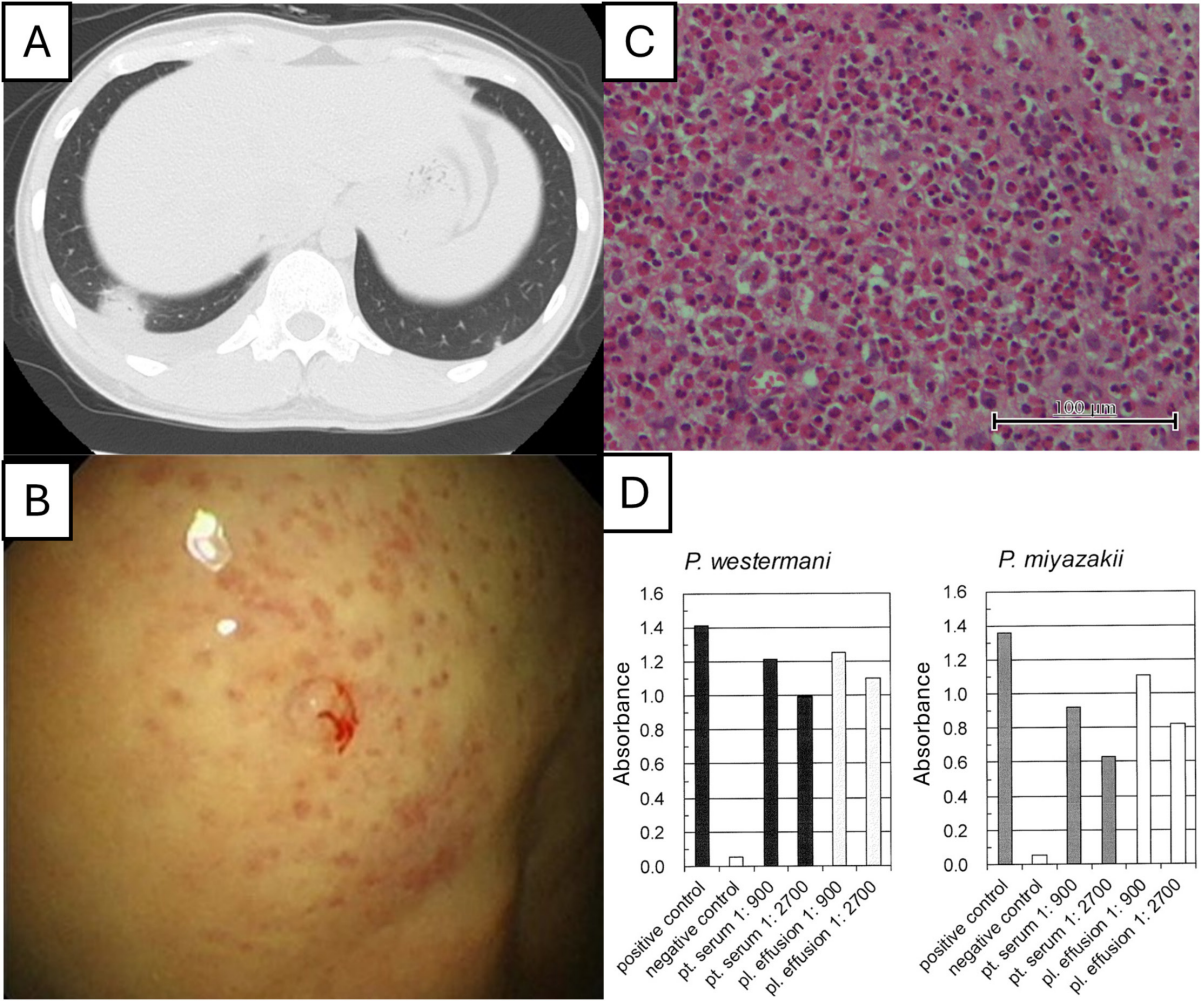
High‐resolution computed tomography (HRCT) images of Case 2, thoracoscopic and pathological findings, and results of antiparasitic antibody testing (microplate enzyme‐linked immunosorbent assay). (A) HRCT image taken at the initial visit to the referring physician, showing right‐sided pleural effusion and nodules in the right lower lobe. (B) Thoracoscopic findings revealing a whitish, thickened visceral pleura with scattered erythematous spots, along with small, elevated lesions. (C) Haematoxylin and eosin staining at high magnification (original magnification, 400 ×) revealing marked eosinophilic infiltration in the pleural biopsy specimen. (D) Pretreatment serum and pleural effusion antiparasitic antibody tests were positive for Paragonimus westermani and *P. skrjabini* miyazakii.

A 24‐year‐old Japanese man presented to a local hospital with a 2‐week history of right‐sided chest pain. HRCT revealed a nodular lesion in the right lower lobe with associated right‐sided pleural effusion (Figure 2A). Consequently, bacterial pneumonia with parapneumonic effusion was suspected, and levofloxacin was administered. However, the patient deteriorated and showed increased pleural effusion. He was subsequently referred to our hospital.

Blood tests showed peripheral eosinophilia (2540/μL) and elevated serum total IgE levels (853 IU/mL). Other laboratory findings were unremarkable. HRCT revealed a further increase in right pleural effusion. Detailed history‐taking revealed that the patient had consumed previously frozen freshwater crabs (*E. japonica*) at the same restaurant as Case 1, 3 months before the onset of symptoms. Stool and sputum examinations were negative for parasite eggs.

Thoracoscopy was performed under local anaesthesia, revealing a whitish, thickened visceral pleura with scattered erythematous spots, along with small, elevated lesions. Biopsy specimens were obtained from these sites (Figure 2B). Pleural fluid was pale yellow and turbid, with eosinophil‐predominant cellularity (cells: 14,300/μL; eosinophil: 78.0%), elevated lactate dehydrogenase levels (799 U/L) and decreased glucose levels (29 mg/dL). Pleural fluid cytology was graded as Class II, and no parasite eggs were detected. Histopathological examination of the pleural biopsy specimens revealed marked eosinophilic infiltration (Figure 2C); however, no granulomas or parasite eggs were identified.

Antiparasitic antibody testing of serum and pleural fluid using multiple‐dot ELISA was positive for *P. westermani* and *P. skrjabini miyazakii*. Based on the patient's dietary history and microplate ELISA findings, pulmonary paragonimiasis was diagnosed (Figure 2D). Treatment with praziquantel (75 mg/kg/day for 3 days) was initiated, leading to resolution of eosinophilia and pleural effusion, with no recurrence.

## 3 Discussion

The present cases demonstrate that paragonimiasis can occur following the consumption of frozen freshwater crabs (*E. japonica*), with clinical manifestations varying significantly among individuals exposed to the same source.

Paragonimiasis remains endemic in regions where freshwater crab consumption is prevalent, especially in Asia. In Japan, the majority of cases are reported in Kyushu. Infections in foreign patients are mainly attributed to the ingestion of raw freshwater crabs, whereas approximately half of domestic cases are linked to the consumption of raw wild boar or deer meat [1].

Effective prevention of paragonimiasis includes thoroughly cooking freshwater crabs harbouring metacercariae (at 55°C for 5 min) or freezing them (at −18°C for 100 min or at −20°C for 48 h). Furthermore, prolonged soaking in soy sauce with a high salt concentration can also inactivate the parasites (for 64 days in soy sauce containing ≥ 5% NaCl, 30 days in 10% NaCl soy sauce and 14 days in 20% NaCl soy sauce) [2]. Cases have recently increased across the country, likely due to expanded frozen food distribution, increasing popularity of ethnic delicacies, such as Chinese drunken crab and Korean gejang, and an increase in foreign residents [3].

In the present cases, the crabs had originated from Hokkaido, where a few cases of paragonimiasis have been reported, and were reportedly frozen at −20°C for over 1 month at the restaurant, thereby meeting the recommended inactivation criteria [2]. To our knowledge, this is the first report indicating that frozen freshwater crabs may still pose a risk of paragonimiasis. However, alternative explanations cannot be entirely ruled out, such as inadequate temperature control or cross‐contamination from deer meat handled at the same restaurant [1].

Definitive diagnosis relies on the identification of *Paragonimus* eggs; however, the detection rate in sputum and stool specimens is low, and diagnostic accuracy varies depending on the method used. Bronchoscopy or thoracoscopy may improve detection in pulmonary cases [4]. Peripheral eosinophilia and elevated serum IgE levels are common, and serological testing is particularly useful when eggs are not detected [1], as in our reported cases.

In outbreaks linked to freshwater crabs, patients may present with a wide range of symptoms, including pulmonary and extrapulmonary manifestations [1, 5]. Although the two patients in the current report were of the same age and consumed the same previously frozen crabs from the same restaurant on the same day, the timing of symptom onset differed. There was no history of consuming raw freshwater crabs or raw wild boar or deer meat other than the freshwater crabs described in this report. Initial HRCT showed peripheral airspace consolidation mimicking CEP in Case 1, whereas pleural effusion with nodules was noted in Case 2. Paragonimiasis typically develops within 2–8 weeks after ingestion of infected freshwater crabs, although clinical symptoms may appear several weeks to a few months later. Such clinical diversity may be attributed to differences in parasite ploidy and migration routes [5].

The current cases demonstrate that paragonimiasis can result from the consumption of frozen freshwater crabs (*E. japonica*) and that the clinical presentations may vary, even among individuals consuming the same crabs. As a limitation, possibilities such as inadequate temperature control or cross‐contamination from deer meat via chopping boards or knives cannot be excluded. In patients with peripheral eosinophilia, parasitic infections such as paragonimiasis should be considered, and a detailed dietary history is essential. Furthermore, proper parasite inactivation methods must be employed during the preparation of freshwater crustaceans and raw wild boar or deer meat. Raising awareness of these preventive measures should be promoted, not only in restaurants but also among the general public.

## Author Contributions

All listed authors contributed equally to the article.

## Funding

The authors have nothing to report.

## Consent

The authors declare that written informed consent was obtained for the publication of this manuscript and its accompanying images, and attest that the consent form used complies with the journal requirements as outlined in the author guidelines.

## Conflicts of Interest

The authors declare no conflicts of interest.

## Data Availability

Data sharing not applicable to this article as no datasets were generated or analysed during the current study.
